# Quality of Life: A Longitudinal Analysis of Correlates of Morale in Old Age

**DOI:** 10.1100/tsw.2004.9

**Published:** 2004-03-03

**Authors:** Said Shahtahmasebi

**Affiliations:** School of Mathematics and Statistics, Faculty of Health and Sciences, Christchurch Polytechnic Institute of Technology, PO Box 540, Christchurch, New Zealand

**Keywords:** heterogeneity, past behaviour, depression, quality of life

## Abstract

This paper examines recurrent continuous morale in old age within a statistical modelling paradigm. The Anglicised Philadelphia Geriatric Centre Morale Scale was used as a small component of a major longitudinal study of old age in rural North Wales, U.K. The literature review and cross-sectional analysis of morale in old age is published elsewhere. This paper deals with the aspect of the longitudinal analysis of morale in old age. The proposed statistical modelling relates recurrent morale to a set of explanatory variables that includes subjective as well as objective measures. In order to assess the degree to which explanatory variables influence morale, an adequate statistical model must handle the possibility that substantial variation between respondents will be due to unmeasured and potentially unmeasurable variables (residual heterogeneity), multicollinearity, and past behaviour effect. These applications are illustrated using morale in old age from the North Wales Longitudinal Study Old Age. The results suggested a strong presence of heterogeneity effect, i.e., current levels of morale appear to be individual specific and independent of its previous levels.

## INTRODUCTION

It is suggested that given increasing focus on chronic disease by geriatric medicine, attention to morale is an important strategy for maximizing quality of life[1]. Sullivan[1] further suggested that improvement in the care of the elderly may be possible by getting physicians to detect and treat problems in morale in old age. The literature reports a number of variables as correlates of morale in old age such as health, income, community activities, mobility, and level of social support[2]. The relationship between morale in old age, depression, and dementia is also well documented[3,4,5,6]. In practice, depression in old age is common, underdiagnosed, and undertreated[7,8,9,10,11] and poor prognosis is associated with coexisting dementia and ill health[6]. Depression in turn is related to reported health complaints[12], pain[13], and income[14]. Thus, there may be a true underlying relationship between morale and variables reported in the literature, however, there is a lack of clarity as to how these variables interact over time and what impact they have on morale. Indeed, in a series of papers, Ventegodt and colleagues[15,16,17,18,19,20] put forward a complex and sophisticated theoretical and philosophical framework for scientific investigation of quality of life. They suggest a global quality of life measure incorporating aspects such as perception and attitudes, well being, satisfaction with life, happiness, meaning of life, the biological information system, realizing life potential, fulfillment of needs, and objective factors. Most of these aspects are processes themselves and therefore dynamic with temporal dependencies within and between the processes. For example, changes in morale may be due to changes in individuals (e.g., increased frailty) or it may be due to constancy within individuals (e.g., personality); morale may return to its previous levels following an intervention or an event.

One of the fundamental methodological problems, quite apart from the issues of defining and measuring quality of life, is disentangling the complex inter-relationships between quality of life and other variables. In reviewing the literature, Wenger[2] emphasized the difficulties inherent in attempting to disentangle the relationships of interest. As with any research based on survey data, it is difficult to distinguish systematic from random patterns due to other variables. The flexibility of survey/observational studies to measure a wide range of subjective and objective variables adds complexities to analyses. Survey studies of elderly people living in the community do not often include measures of depression, morale, or dementia all at the same time. In this context, quite apart from the methodological issues of multicollinearity and the direction of causality, the issue of bias in the sample due to unmeasured or omitted variables needs also to be addressed. Wenger et al.[21] have addressed some of the difficulties associated with the analysis of survey data and reported an improved cross-sectional multivariate analysis to incorporate two fundamental issues of multicollinearity and the direction of causality. However, cross-sectional analysis does not allow an assessment of past behaviour (e.g., state dependence) and does not handle heterogeneity effect due to omitted variables (e.g., frailty).

Heterogeneity results when systematic but unmeasured characteristics of individuals contribute to response pattern over time. In survey studies, some individual characteristics are often omitted from the study either because they are unobserved or difficult to measure. Omitted characteristics, such as frailty, could lead to spurious relationships between the observed characteristics and the outcome variable. Past behaviour effect exists when the experience of a particular outcome itself changes the probability of experiencing that event on subsequent occasions. Again, it is clear that past behaviour cannot be addressed within cross-sectional designs. Heterogeneity and past behaviour effects are common when dealing with behavioural data and ignoring them will produce bias in variability estimates and tests of treatment related hypothesis[22,23].

Longitudinal studies provide an additional flexibility to increase control in the analysis; with panel data, it is possible to control for heterogeneity, temporal dependencies, and to get a better understanding of the direction of causality[22,24]. However, the traditional methods of analysing longitudinal data such as analysis of end points and analysis of variance do not fully utilise the properties of the data; in particular, such methods exclude the heterogeneity effect[23,25]. Increasing numbers of methods are available to improve cross-sectional studies. While these methods tend to reduce the number of variables to focus on, they can raise other complex questions about the nature of the process over time. Such outcomes are, in their own right, an important aspect of the results.

A review of the literature on morale in old age is not attempted, the interested reader is referred to Wenger[2,21]. In this paper, a method of analysing longitudinal data is demonstrated, which allows for heterogeneity *simultaneously* with multicollinearity and past behaviour effects. For comparison purposes, a cross-sectional analysis of the same data is also presented. These applications are illustrated using repeated observations on morale in old age.

## METHODS

The data come from a survey of elderly people living in rural North Wales[21]. The data structure is shown in Table 1A. As can be seen, three time data were available only for the old elderly (respondent who were 75+ in 1979). Furthermore, a variable of interest (the Pfeiffer dependency scores) was included in the questionnaire in 1983 and 1987 interval points. These scores are interviewers' observed health and dependency using five scales (see Appendix I), which may be seen as more objective than self-assessed state of health and other self-reported dependencies. Therefore, it was decided to restrict the analysis to the 1983 and 1987 time points. This strategy also allows the flexibility to test the past behaviour effect using data from 1979.

**TABLE 1A**
**The Structure of the Longitudinal Data**

| 1979 | 1983 | 1987 |
|---|---|---|
| 534 respondents (75+) were interviewed | First follow-up of the sample | Second follow-up of the sample |
| | 108 old elderly reinterviewed | All survivors reinterviewed |
| | 117 deceased | 107 deceased since 1983 |
| | 17 in residential care | 29 in residential care |

**TABLE 1B**
**Revised Philadelphia Geriatric Centre Scale**

| | Yes | No | D/K |
|---|---|---|---|
| Do things keep getting worse as you get older? | 1 | 3 | 2 |
| Do you have as much energy as you did last year? | 3 | 1 | 2 |
| Do you feel lonely much? | 1 | 3 | 2 |
| Do you see enough of your friends and relatives? | 3 | 1 | 2 |
| Do little things bother you more this year? | 1 | 3 | 2 |
| As you get older, do you feel less useful? | 1 | 3 | 2 |
| Do you sometimes worry so much you can't sleep? | 1 | 3 | 2 |
| As you get older, are things better than expected? | 3 | 1 | 2 |
| Do you sometimes feel life isn't worth living? | 1 | 3 | 2 |
| Are you as happy now as when you were younger? | 3 | 1 | 2 |
| Do you have a lot to be sad about? | 1 | 3 | 2 |
| Are you afraid of a lot of things? | 1 | 3 | 2 |
| Do you get angry more than you used to? | 1 | 3 | 2 |
| Is life hard for you most of the time? | 1 | 3 | 2 |
| Are you satisfied with your life today? | 3 | 1 | 2 |
| Do you take things hard? | 1 | 3 | 2 |
| Do you get upset easily? | 1 | 3 | 2 |

## Morale Scale

The Philadelphia Geriatric Centre Morale Scale[26] was Anglicised and adapted (see Table 1B) as part of a major study of old age in rural North Wales[21,27]. This scale was used for a number of reasons: (1) it has been widely used and therefore maximises replication, (2) it has been shown to have a high correlation with the judgment of clinical psychologists[28], (3) it has been shown to be reliable with very old respondents and with rural populations, and (4) it was the only available scale at the time the study began which includes social relationships among its items[27]. Lawton[28] reported a satisfactory reliability and high internal consistency. On the basis of piloting and in order to extend the range of possible response, which Lawton felt to be desirable ([28], p. 160), "don't know" was added to the yes/no option. A need was also felt for an aggregate measure of morale, which Lawton identified as sufficient for many research purposes ([28], p.164). High morale item responses were therefore scored as 3 and low morale responses as 1. Those whose morale was insufficiently high or low to give an unequivocal response scored 2 as an intermediate measure as shown in the table. Aggregate morale was then calculated as an average of the 17 responses. This aggregate measure formed continuum from 1.0 (low morale) to 3.0 (high morale).

There were 38 cases with a valid morale score at each interval point 1983 and 1987 in the North Wales Elderly Data. The sample size appear small, however Table 1C suggests that it is sufficiently large to detect a small difference of 0.1 on the morale scale with 5 and 20% probability of type I and type II error (power of 80%), respectively[29]. Because of the relatively small sample size, large categorical variables were collapsed into two or three categorical variables. The list of explanatory variables is shown in Appendix I.

**TABLE 1C**
**Sample Size Required to Yield a Power of 80% to Detect a Difference of 0.1 on the Morale Scale at Two Time Points, Where ρ is the Corr[$y_{i,t}$,$y_{i,t-1}$] and α is the Probability of Type I Error**

| α | P = 80% | d = 0.1 | ρ | $\sigma^2$ | | | |
|---|---|---|---|---|---|---|---|
| | | | | 0.5 | 1.0 | 1.5 | 2.0 |
| 5% | | | 0.2 | 20 | 40 | 59 | 79 |
| | | | 0.4 | 15 | 30 | 45 | 59 |
| | | | 0.6 | 10 | 20 | 30 | 40 |
| 10% | | | 0.2 | 16 | 31 | 47 | 62 |
| | | | 0.4 | 12 | 24 | 35 | 47 |
| | | | 0.6 | 8 | 16 | 24 | 31 |

## Models for Analysis

A regression type model was specified with morale as the response variable. The advantages of a regression model over other methods, such as analysis of variance or end-point, are the ability to handle heterogeneity as well as a large number of continuous and categorical explanatory variables, to provide an estimate of the difference in the pattern of response given an explanatory variable, and to allow the estimate of not only the main effect of explanatory variables, but also first and higher order interactions between them.

For the longitudinal analysis of morale, the 1983 and 1987 data points were used, thus forming a sequence of observations on the same individuals. Fitting the conventional regression model to such data led to a well-known specification error. The regression model was modified with a "variance component"

specification in which the heterogeneity effect is implicitly defined in the equation as a time constant term $\{\theta\}$ separately from the regression error term $\{\varepsilon\}$[30] (see Appendix II). There are two methods of model estimation for models with two unobserved components: the conditional likelihood method and the marginal likelihood method. The conditional likelihood method, sometimes referred to as the difference method, eliminates $\theta$ by subtracting the response equation for time 2 from that of time 1. This differencing leads to the term $\theta$ and all other constant variables (such as sex) to be eliminated from the model, thus restricting inference to time varying variables. The resulting model could then be fitted using the likelihood or least squares method[31,32,33]. A drawback of this method is the loss of information. However, the method is easy to adopt and apply in particular when dealing with continuous response variable measured at two time points. Furthermore, loss of information can be compensated for by integrating the instrumental variables method in the statistical modelling[34].

In this paper, the marginal likelihood method was used. This method, also referred to as the integrated likelihood method, eliminates $\theta$ by integrating it out of the likelihood equation[30,35]. This method allows time varying and time constant variables in the analysis. Notice that the independence assumption, that the error terms are independent of the included explanatory variables, must still hold. This method provides a useful statistic for the heterogeneity effect that is given by the within-individual correlation ($R^2$)(see Appendix II).

The forward substitution approach was employed to model building because of the large number of explanatory variables. The computer program VARCL[36], which handles variance component models with a continuous response variable, was used for model fitting. Variables were tested in the model one at a time using the likelihood ratio ($\chi^2$) and the F-ratio goodness of fit test, for the longitudinal and the pooled cross-sectional methods, respectively. The variable with the largest $\chi^2$ and associated smallest *p*-value was entered into the model next. The process was repeated with those variables, which were significant at 10% level, until there were no statistically significant variables remaining.

For comparison purposes, a cross-sectional analysis of the same data was also carried out. The standard regression model without structural modification was fitted to the data. Thus, the cross-sectional model assumes that the observations on the same individuals in 1983 are independent of the 1987, i.e., giving a total sample of ($2 \times 38$) 76 cases.

Some authors have suggested that subjective variables may play an intervening role between objective variables and morale[2]. Variables were classified as subjective if they were subject to the respondent's interpretation[21], see Appendix I. Through their possible relationship with the omitted variables, the subjective variables are responsible for model mis-specification and erroneous results. As described in the methods section above, the longitudinal modelling approach allows control for the omitted variables.

## RESULTS AND DISCUSSION

Past behaviour effect was tested within the modelling framework using dummy variables representing morale in 1979 and 1983 in the longitudinal analyses. For this data set, past levels of morale do not appear to be correlated with the present levels of morale. The results from the different methods are summarised in Table 2.

The cross-sectional method suffers from a lack of clarity due to a marginal choice between the variables "confidant" ($p = 0.003$) and "Pfeiffer mental rating" ($p = 0.02$) (one is a more limited and subjective manifestation of the other), "confidant" is selected to be included in the final model. The fitting process for the longitudinal model clearly reveals that including the cumulative rating in the model removes the high significance of both physical and mental rating, i.e., cumulative Pfeiffer dependency appears to control for the effect of both physical and mental ratings.

The results from the cross-sectional method suggest that dependency due to physical health (as measured by the Pfeiffer Scale) may lead to lower levels of morale; on average, those highly dependent individuals as shown by the physical dependency scale score lower on the morale scale. Perception of having a "confidant" appears to be positively correlated with morale; availability of "confidant" increases levels of morale as opposed to not having a "confidant" at all. It is plausible that perceived companionship in old age as reflected by "confidant" operates in the opposite direction of the effects from any lack of quality of life associated with lowered mental competence that may be affecting morale.

**TABLE 2**
**Summary of Results from Pooled Cross-Sectional and Longitudinal Analysis of Morale**

| Explanatory Variables | Cross-Sectional (Pooled) | | Longitudinal (Marginal) | |
|---|---|---|---|---|
| | p.e | s.e | p.e | s.e |
| | 1 | | 2 | |
| **Objective variables** | | | | |
| Pfeiffer - physical rating | –0.18 | 0.04 | | |
| Pfeiffer - cumulative | | | –0.05 | 0.01 |
| **Subjective variables** | | | | |
| Confidant | | | | |
| No one | 0.00 | | | |
| Spouse | 0.54 | 0.15 | | |
| Other | 0.32 | 0.13 | | |

**TABLE 3**
**Estimate of Variance Components (Longitudinal Model)**

| | Variance | Sigma | Standard error for Sigma |
|---|---|---|---|
| Intervals ($\varepsilon_{it}$) | 0.04 | 0.21 | |
| Individual ($\theta_i$) | 0.08 | 0.28 | 0.04 |
| Deviance | 43.00 | | |

$$R^2 = \frac{(\sigma_\theta)^2}{(\sigma_\theta)^2 + (\sigma_\varepsilon)^2} = \frac{0.08}{0.08 + 0.04} = 0.66$$

The results from the marginal likelihood method, allowing control for heterogeneity effect, seem to suggest no direct link between subjective variables and morale for this data set. The objective variable "cumulative Pfeiffer" dependency measure appears to control for the effect of all the other variables. The high within-individual correlation ($R^2$ = 0.66, Table 3) emphasizes the importance of adopting a methodology that allows control for heterogeneity effect. The results from this model suggest that those with higher physical health dependency, as measured by the cumulative Pfeiffer score, on average, score 0.25 (5*-0.05) lower on the morale scale (other characteristics being the same). The within-individual correlation of ($R^2$ = 0.66) confirms the substantial heterogeneity effect in the data which means that temporal dependencies of morale in old age is due to heterogeneity rather than previous state of morale, i.e., those who currently appear to be of low morale are inherently so. This result highlights the important role of panel data and methodologies that allow control for this effect.

## CONCLUSION

It is reassuring to note that, given the size of the sample, the results appear robust, which is indicated by the small parameter estimates and their standard errors. Furthermore, the model fitting approach of excluding and including subjective variables ensured the identification of the weaker effects in the data, as well as providing an idea about the role of subjective variables that is confirmed by the longitudinal modelling. On the whole, there were fewer variables to focus on than reported in the literature.

Clearly, it is important to employ panel data and methodologies that handle the heterogeneity effect. On the other hand, ignoring heterogeneity effect in the analysis may lead to spurious relationships and an overestimation of the effect of included explanatory variables. For example, while age and state of health may appear to be significantly related to morale in old age, such relationships may well be due to a correlation between age, health with depression, and frailty, which are omitted from the study. This result has implications for social and health policy development and policy evaluation. In an era where there is a heavy emphasis on evidence-based practice, this approach has clear implications for health and social policy development and evaluation. In this context, evidence must be based on relevant and appropriate information.

## ACKNOWLEDGEMENTS

Based on research conducted by Professor Clare Wenger and jointly funded by the Department of Health and Social Security/Welsh Office and Economic and Social Research Council (G00212334). The analysis reported in this paper was carried out during the author's employment at the Centre for Social Policy Research and Development (CSPRD), University of Wales, Bangor.

## BIOSKETCH

**Said Shahtahmasebi** is currently research methodology consultant and statistician at Christchurch Polytechnic Institute of Technology, Christchurch, New Zealand. His work experiences are mainly in the United Kingdom and cover a number of fields including public health, mental health, nursing, operational research, food, nutrition. and gerontology. His area of interests and expertise are related to longitudinal modelling of health related behaviour. He has set up the Good Life Project (http://lists.cpit.ac.nz/thegoodlife/) in New Zealand, which attempts to investigate food holistically.

## APPENDIX I: LIST OF EXPLANATORY VARIABLES

| | |
|---|---|
| **Age** | Continuous |
| **Sex** | Male, female |
| **Income** | *Low*: up to £39, £40–79; *comfortable*: £80–99; *above average*: £100+ |
| **Social class** | Class I, II/III, IV/V |
| **Home tenure** | Owner occupier, local authority rented accommodation, private rented, other (for the old elderly sample; owner occupier, rented, other) |
| **Ethnicity** | Welsh, Non-Welsh |
| **Marital status** | Single, married, widowed, divorced/separated |
| **Social support variables** | |
| Frequency see family | Every day, at least weekly, at least monthly, less often, never/no relatives |
| Relative seen most often | No relatives, child, sibling, other |
| Who cares when ill | Spouse, relative in/out house, friend/neighbour, other, no one/d/k/missing - was used for 1979 cross-sectional analysis only |
| Network type | Family dependent, locally integrated, local self-contained, wider community focused, private restricted[37] |
| **Loss variables** | |
| Change in marital status | No change, lost spouse, married - 1983 and 1987 intervals only |
| Number of years widowed | Continuous |
| **Health** | |
| Housebound | Yes, no |
| Self-assessed health | ***Subjective;*** excellent/good, alright for age, fair/poor |
| Health-limited activities | ***Subjective;*** yes, no |
| **Isolation variables** | |
| Household composition | Lives alone, with spouse, with others |
| Has close relative(s) | Yes, no |
| Visits relatives | At least weekly, at least 6/year, less often, no relatives/never |
| Has telephone | Yes, no |
| Nearest neighbour | Next door attached/det., across road/50–100 yards, 100+ yards |
| Hours spent alone | 0–3 hours/day, 3–9 hours/day, 9+ hours/day |
| Isolation measure | Continuous - a combination of 7 objective variables reflecting social contact[27] |
| **Loneliness variables** | |
| People available to do favours | ***Subjective***; yes, no, never ask favours |
| Presence of real friends | ***Subjective***; yes, no - respondent were asked to list names |
| Wish for more friends | ***Subjective***; yes, no |
| Confidant | ***Subjective***; no one, spouse, other |
| Self-assessed loneliness | ***Subjective***; never/rarely, sometimes, often/always |
| **Pfeiffer score scales *(for 1983 and 1987 only)[38]*** | |

1: Social rating scale (scale 1 = socially active to 5 = inactive)
2: Mental health rating scale (scale 1 = presence of mind to 5 = confused)
3: Physical health rating scale (scale 1 = good health, active to 5 = poor health)
4: Activities of daily living scale (scale 1 = total independence to 5 = total dependence)
5: Economic resources rating scale (scale 1 = financially secure to 5 = insecure)
Cumulative Pfeiffer Score[39]

## APPENDIX II: INTEGRATED OR MARGINAL LIKELIHOOD METHOD

Consider the conventional regression model:

$$\underline{y}_{it} = \underline{\beta x}_{it} + \underline{\varepsilon}_{it}$$

where $\underline{x}$ is the vector of explanatory variables, $\underline{\beta}$ is the vector of the regression parameters, and $\underline{\varepsilon}$ is the vector of i.i.d regression error. Longitudinal data structure form a hierarchy of clusters, i.e., individual i at time $t_1$, $t_2$,..., $T_N$ form cluster i which will be more similar than those in other clusters. When applying the conventional regression model, the equation above violates the independence assumption; explanatory variables $\underline{x}$ may be correlated with the i.i.d error $\underline{\varepsilon}$ due to observations on the same individual in each cluster. To some extent, this problem can be overcome by adopting a "variance component" specification, including an individual specific error term into the equation[30] as follows:

*for time t* $\quad \underline{y}_{it} = \underline{\beta x}_{it} + \underline{\theta}_i + \underline{\varepsilon}_{it}$

*for time t+1* $\quad \underline{y}_{it+1} = \underline{\beta x}_{it+1} + \underline{\theta}_i + \underline{\varepsilon}_{it+1}$

where $\underline{\theta}$ is the vector of additional individual specific error or residual heterogeneity. The error terms are assumed i.i.d and to be independent of the $\underline{x}$. Notice that the nuisance parameter $\theta$ is time constant. With the longitudinal approach, past behaviour could easily be introduced as the sequence of response observed at the previous time point $y_{it-1}$ as follows:

*for time t* $\quad \underline{y}_{it} = \{\underline{\alpha y}_{it-1}\} + \underline{\beta x}_{it} + \underline{\theta}_i + \underline{\varepsilon}_{it}$

*for time t+1* $\quad \underline{y}_{it+1} = \{\underline{\alpha y}_{it}\} + \underline{\beta x}_{it+1} + \underline{\theta}_i + \underline{\varepsilon}_{it+1}$

To operationalize this model, we must eliminate $\theta$ before model fitting because it is not possible to estimate the nuisance parameter simultaneously with the structural parameters (see [30,35,40]).

The marginal likelihood method eliminates $\theta$ by integrating it out of the likelihood. The likelihood is based on the probability or density unconditional on $\theta$, given by:

$$f(S_i \,|\varphi;x_i) = \int f(S_i \,|\beta;x_i;\theta)\, f(\theta)\, d\theta$$

where S is the observed sequence of outcome and $f(\theta)$ is the p.d.f of $\theta$, and the parameter vector $\varphi$ consists of $\beta$ and parameters of $f(\theta)$. Therefore each case contributes to the likelihood:

$$L_i(\varphi) = \int L_i(\beta, \theta)\, df(\theta).$$

For a sample of N individuals (with up to T times observations), the integrated or marginal log-likelihood is thus given by:

$$l = \sum_{i=1}^{N} \log L_i(\varepsilon) = \sum_{i=1}^{N} \log[f(S_i \mid \phi; x_i)]$$

The integration will lead to elimination of nuisance term $\theta$ from the model[35]. The individual specific term $\theta_i$ and the i.i.d error term $\varepsilon_{it}$ (between intervals) are assumed to be normally distributed with zero mean, unknown variances $\sigma^2_\theta$ and $\sigma^2_\varepsilon$, respectively, and zero covariances. It can be deduced that the intra-individual correlation is given by[30]:

$$R^2 = \frac{(\sigma_\theta)^2}{(\sigma_\theta)^2 + (\sigma_\varepsilon)^2}$$

For model selection and model fitting VARCL[36] was used.
